# Fasting-Induced Changes in Hepatic P450 Mediated Drug Metabolism Are Largely Independent of the Constitutive Androstane Receptor CAR

**DOI:** 10.1371/journal.pone.0159552

**Published:** 2016-07-19

**Authors:** E. M. de Vries, L. A. Lammers, R. Achterbergh, H-J Klümpen, R. A. A. Mathot, A. Boelen, J. A. Romijn

**Affiliations:** 1 Department of Medicine, Academic Medical Center, Meibergdreef 9, 1105 AZ, Amsterdam, the Netherlands; 2 Department of Hospital Pharmacy, Academic Medical Center, Meibergdreef 9, 1105 AZ, Amsterdam, the Netherlands; 3 Department of Medical Oncology, Academic Medical Center, Meibergdreef 9, 1105 AZ, Amsterdam, the Netherlands; 4 Department of Endocrinology and Metabolism, Academic Medical Center, Meibergdreef 9, 1105 AZ, Amsterdam, the Netherlands; The University of Iowa, UNITED STATES

## Abstract

**Introduction:**

Hepatic drug metabolism by cytochrome P450 enzymes is altered by the nutritional status of patients. The expression of P450 enzymes is partly regulated by the constitutive androstane receptor (CAR). Fasting regulates the expression of both P450 enzymes and CAR and affects hepatic drug clearance. We hypothesized that the fasting-induced alterations in P450 mediated drug clearance are mediated by CAR.

**Methods:**

To investigate this we used a drug cocktail validated in humans consisting of five widely prescribed drugs as probes for specific P450 enzymes: caffeine (CYP1A2), metoprolol (CYP2D6), omeprazole (CYP2C19), midazolam (CYP3A4) and *s*-warfarin (CYP2C9). This cocktail was administered to wild type (WT, C57Bl/6) mice or mice deficient for CAR (CAR^-/-^) that were either fed ad libitum or fasted for 24 hours. Blood was sampled at predefined intervals and drug concentrations were measured as well as hepatic mRNA expression of homologous/orthologous P450 enzymes (Cyp1a2, Cyp2d22, Cyp3a11, Cyp2c37, Cyp2c38 and Cyp2c65).

**Results:**

Fasting decreased Cyp1a2 and Cyp2d22 expression and increased Cyp3a11 and Cyp2c38 expression in both WT and CAR^-/-^ mice. The decrease in Cyp1a2 was diminished in CAR^-/-^ in comparison with WT mice. Basal Cyp2c37 expression was lower in CAR^-/-^ compared to WT mice. Fasting decreased the clearance of all drugs tested in both WT and CAR^-/-^ mice. The absence of CAR was associated with an decrease in the clearance of omeprazole, metoprolol and midazolam in fed mice. The fasting-induced reduction in clearance of *s*-warfarin was greater in WT than in CAR^-/-^. The changes in drug clearance correlated with the expression pattern of the specific P450 enzymes in case of Cyp1a2-caffeine and Cyp2c37-omeprazole.

**Conclusion:**

We conclude that CAR is important for hepatic clearance of several widely prescribed drugs metabolized by P450 enzymes. However the fasting-induced alterations in P450 mediated drug clearance are largely independent of CAR.

## Introduction

The nutritional status modulates hepatic drug metabolism in experimental models and in patients [1]. This may affect efficacy and safety of drugs with a narrow therapeutic window that are extensively metabolised by the liver. Cytochrome P450 enzymes (P450) are important in the homeostatic control of different endobiotics (including bile acids and steroids) and the detoxification of xenobiotics, including drugs. Nutritional status is an important factor influencing the expression of these P450 enzymes. For instance, 36h fasting differentially affects the hepatic expression of different P450 expression in rats. In addition, 36h fasting in humans increases the oral caffeine and decreases oral *s-*warfarin clearance, in accordance with the differentially regulated P450 enzymes [2].

Expression of constitutive P450 enzymes is regulated mainly by basal transcription factors to maintain adequate gene expression. P450 induction by xenobiotics is regulated by nuclear receptors such as the pregnane X receptor (PXR) and the constitutive androstane receptor (CAR) [3]. Fasting can interfere with both pathways and thus affect both constitutive and induced expression of P450 enzymes. A well-known PXR target gene is CYP3A4, the CYP-isoform estimated to account for approximately 30% of all hepatic drug metabolism in humans [4]. A role for CAR in xenobiotic metabolism was first discovered when CAR was identified as the nuclear receptor important for induction of CYP2B2 by phenobarbital [5]. More interest was raised in the role of CAR in drug metabolism by the observation that CAR deficient mice showed less acetaminophen induced hepatotoxicity [6]. Furthermore, it has been shown recently that a large amount of P450 enzymes is regulated by CAR, exceeding the amount of PXR regulated P450 enzymes [7].

CAR expression is increased upon fasting in the liver in rats [2] and mice [8]. This fasting-induced increase in CAR is regulated by a complex interplay with other nuclear receptors that are sensitive to nutritional status such as PPARα, HNF4α and PCG-1α [8, 9]. The finding that fasting increases CAR expression and alters the clearance of orally administered drugs prompted us to hypothesize that CAR mediates the fasting-induced alterations in hepatic clearance of several drugs. In order to test this hypothesis we used a P450 probe-drug cocktail validated in humans [10] that was administered to fasting and ad libitum fed mice that were deficient for CAR (CAR^-/-^ mice) and compared the exposure of the specific drugs of this cocktail to the exposure observed in fed and fasted wild type (WT) mice.

The drugs in the cocktail are validated probes for five P450 enzymes which play an important role in drug metabolism in humans: midazolam (CYP3A4), caffeine (CYP1A2), S-warfarin (CYP2C9), omeprazole (CYP2C19) and metoprolol (CYP2D6). To gain insight in the role of CAR in fasting induced alterations in P450 enzymes, we also studied changes in mRNA expression of P450 enzymes in mice that correspond to the five P450 enzymes in humans: Cyp3a11, Cyp1a2, Cyp2c37, Cyp2c38 and Cyp2d22 respectively [11]. Cyp1a2 shows high homology between human and mice. In addition, Cyp1a2 has been reported to be the P450 isoform primarily responsible for caffeine clearance in mice [12]. In mice, the Cyp2c family is even more complex than in humans. We therefore studied the three most abundant Cyp2c isoforms in mouse liver (Cyp2c37, Cyp2c38 and Cyp2c65) [11]. Like in humans, midazolam is a Cyp3a substrate in mice, although Cyp2c is also able to metabolize midazolam in mice but with much lower turnover rates than Cyp3a enzymes [13]. Of the mouse Cyp3a isoforms, Cyp3a11 is the suspected ortholog to human CYP3A4 and is highly expressed in murine liver [11]. Cyp2d22 is the suggested murine ortholog of human CYP2D6 [14]. In addition to Cyp3a11, which is a prototypical PXR target gene, all of these P450 isoforms are regulated by CAR [7]. This experimental design enabled us to answer three research questions: 1) What are the effects of fasting on P450 mRNA expression in naïve (saline injected) WT and CAR^-/-^ mice? 2) Does the substrate inducibility of P450 mRNA expression by injection of the drug cocktail differ between WT and CAR^-/-^ mice? 3) Are the fasting-induced changes in drug clearance mediated by CAR?

## Materials and Methods

### Animals

Female CAR^-/-^ (C57BL/6-*Nr1i3*^*tm1*.*1Arte*^) and WT (C57BL/6NTac) mice (*mus musculus*) (9–11 weeks old) were used in this study (Taconic Biosciences, Hudson, NY, USA). Male mice tend to fight more than female mice when housed in groups. Since previous studies have shown that infections can also affect P450 enzyme activity, we used female mice to exclude any effects of littermate-inflicted wounds that could confound our results. Mice were housed in a 12 hour light/dark cycle and were provided with standard laboratory chow (CRM (E) chow from Special Diet Services, Essex, UK). Environmental enrichment was present in the form of a cardboard nest box. On day one of the experiment, food was removed in the morning from the fasting groups whereas the fed groups received food ad libitum. The following day, after 20 hours of fasting or feeding, the drug cocktail or saline was administered by intraperitoneal (i.p.) injection (n = 6 per group). Blood was sampled (50 μl per sample at each timepoint) from the tail vein at t = 15 minutes, t = 30 min, t = 1h, 2h. Because of limitations in blood volume that we could sample, no t = 0 sample was obtained. Serum of saline injected mice served as a control. At t = 4 h after i.p. injection, the mice were sacrificed by heart puncture under isoflurane anaesthesia bringing the total time on the fasting or feeding regimen to 24 hours. Serum was stored at -80°C. The liver was dissected, snap frozen in liquid nitrogen and stored in -80°C until further analysis. The study was approved by the local animal welfare committee of the Academic Medical Center Amsterdam (DEC, permit number: DIE103025) and carried out in accordance to local rules and regulations. Animal welfare and physical condition were monitored daily. A humane endpoint protocol was in place, however no animals became severely ill or died prior to the experimental endpoint.

### Drug cocktail

The validated drug cocktail has been described before [10], and the doses were adjusted for the average body weight of mice (20 gram). Per 20 gram BW, 0.5 ml was injected intraperitoneally consisting of midazolam (5 mg/ml, Actavis, the Netherlands), caffeine (10 mg/ml, VUMC, Amsterdam, The Netherlands), warfarin (5 mg/ml, Radboud UMC, The Netherlands), omeprazole (8 mg/ml, AstraZeneca, The Netherlands) and metoprolol (1 mg/ml, AstraZeneca, The Netherlands).

### RNA isolation and qPCR

Total RNA from mouse liver was isolated using the total tissue RNA isolation kit (Roche Life Science, Indianapolis, IN, USA). RNA concentrations were determined using the Nanodrop (Nanodrop, Wilmington, Delaware USA). The cDNA synthesis was carried out using equal RNA input and the first strand transcriptor cDNA synthesis kit (Roche). Quantitative PCR was performed using the Lightcycler 480 apparatus and the SensiFAST SYBR no-rox kit (Bioline, Londen, UK) and according to the MIQE guidelines. Quantification was performed using the LinReg software [15]. Samples with a mean deviation of more than 5% of the mean efficiency value of the assay were excluded. Calculated values were normalized by the geometric mean of 3 reference gene values (HPRT, Ubiquitin and Cyclophilin) which were selected to be the most stable among different groups. The primers used are displayed in Table 1. P450 enzymes were chosen on the homology with the human counterpart P450 enzymes that are involved in the specific metabolism of the 5 probe drugs [11].

**Table 1:** qPCR conditions. Primer sequences, NCBI accession numbers and annealing temperatures used in the qPCR analysis.

Gene name	Accession number	Primer sequences	Annealing temp.
HPRT	NM_013556	F: 5’-GCAGTACAGCCCCAAAATGG-3’	65
		R: 5’-AACAAAGTCTGGCCTGTATCCAA-3’	
Cyclophilin	NM_011149	F: 5’-GAGACTTCACCAGGGG-3’	65
		R: 5’-CTGTCTGTCTTGGTGCTCTCC-3’	
Ubiquitin	NM_019639	F: 5’-AGCCCAGTGTTACCACCAAG-3’	65
		R: 5’-CTAAGACACCTCCCCCATCA-3’	
CAR (Nr1i3)	NM_009803.5	F: 5’-CGCAGTCCATGCAGGGTTCCA-3’	65
		R: 5’-ACTCCGGGTCTGTCAGGGGA-3’	
PXR (Nr1i2)	NM_010936.3	F: 5’-ACCCTAATGGTGGCTTCCAG-3’	65
		R: 5’-GGTTTGCTGGGCGTTGATG-3’	
Cyp2b10	NM_009999.4	F: 5’-GGACAGTTGCTGTCGTTGAGCCA-3’	65
		R: 5’-GCAGGCGCAAGAACTGACGG-3’	
Cyp1a2	NM_009993	F: 5’-CAGGAGCACTACCAAGACTTCA-3’	65
		R: 5’-TGGATCTTCCTCTGCACGTT-5’	
Cyp2d22	NM_001163472.1	F: 5’-CACCGGTAAAGGTAGCTGGAGT-3’	65
		R: 5’-CCAGCTGTAGGCTGAACAGG-3’	
Cyp3a11	NM_007818	F: 5’-AACCTGGGTGCTCCTAGCAA-3’	65
		R: 5’-AGCAAGGAGAGGCGTTTGAC-3’	
Cyp2c65	NM_028191.2	F: 5’-CAACCCAGAGAAGTTTGACCCCA -3’	65
		R: 5’-CTCTCCCACACAAATCCGTTTTC-3’	
Cyp2c37	NM_010001	F: 5’-GATGGCAATCAACCATTGCAAAA-3’	65
		R: 5’-CTTGCCGATCACATGCTCAA-3’	
Cyp2c38	NM_010002	F: 5’-GGTAGAGAAAACATCCCAATGTCTG-3’	65
		R: 5’-GACATTGCATGGAGCACAGC-3’	

### Bioanalysis of the Cyp450 probe drugs

A liquid chromatography/tandem mass spectrometry (LC-MS/MS) method for the simultaneous determination of plasma concentrations of the five drugs used in the CYP450-probe cocktail was developed and validated [2]. Because of the strict limitation in volume and frequency of blood sampling from mice, we did not take a t = 0 blood sample. However, for all analytes, no significant interfering peaks were detected in matrix blanks.

### Data analysis, presentation and statistical analysis

**Fig 1:**
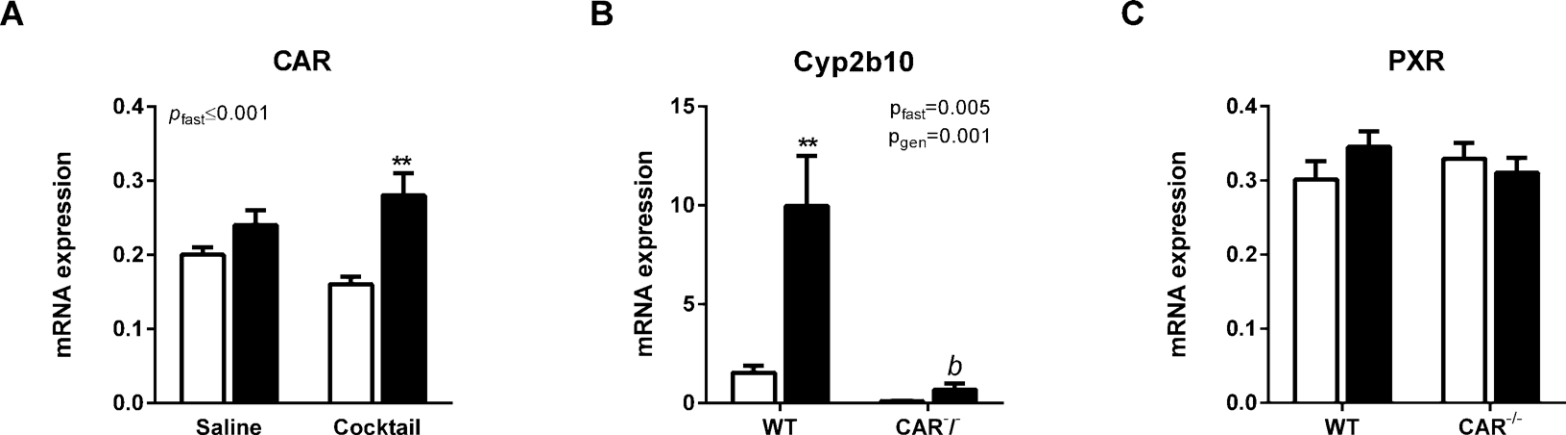
mRNA expression of nuclear receptors CAR and PXR and CAR target gene Cyp2b10. A) CAR mRNA expression in the saline and cocktail injected WT mice. B) Cyp2b10 mRNA expression in saline injected WT and CAR^-/-^ mice. C) PXR mRNA expression in the saline injected WT and CAR^-/-^ mice. Fed mice (white bars), fasted mice (black bars). P-values represent the effect of fasting (*p*_fast_) in the two-way ANOVA (n = 6 per group). Differences between fed and fasted groups as tested by posthoc Student’s t-test are represented by * (** = p≤0.01).

**Fig 2:**
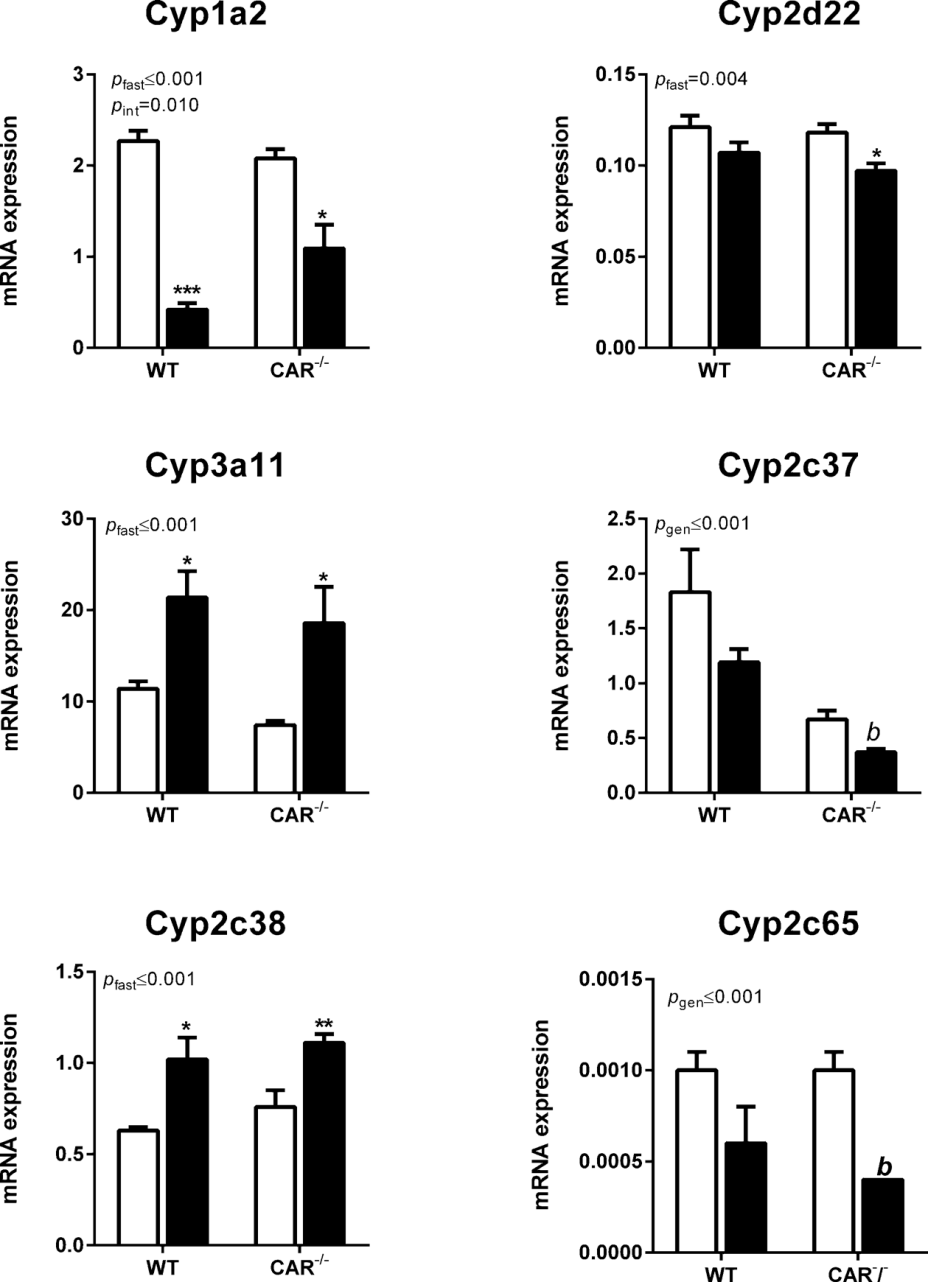
P450 enzyme mRNA expression in the saline injected WT and CAR^-/-^ mice. Fed mice (white bars), fasted mice (black bars). P-values represent the effect of fasting (*p*_fast_), genotype (*p*_gen_) or the interaction of both variables (*p*_int_) in the two-way ANOVA (n = 6 per group). Differences between groups as tested by posthoc Student’s t-test or nonparametric Mann Whitney U test are represented by * in case of the fed vs fasted groups (* = p≤0.05, ** = p≤0.01, *** = p≤0.001) and by ^b^ in case of the WT vs CAR^-/-^ groups (*b =* p≤0.01)

**Fig 3:**
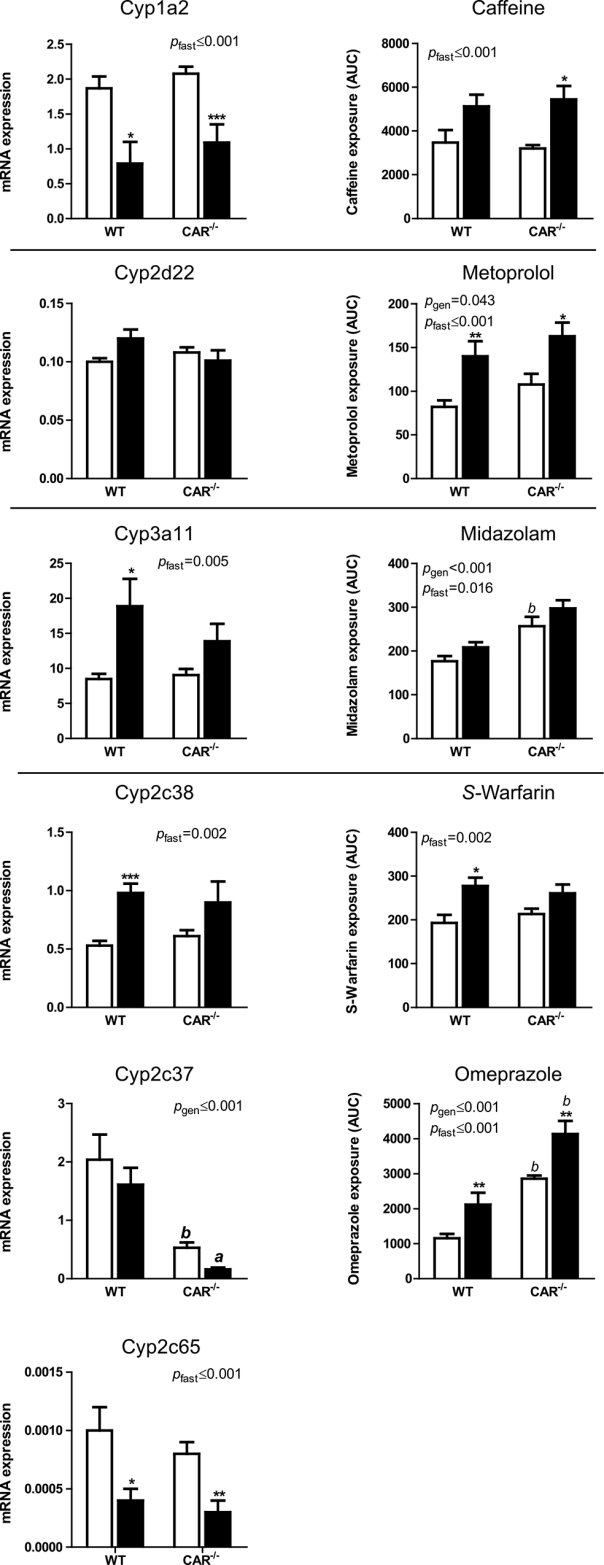
drug clearance and P450 mRNA expression. mRNA expression of the different P450 enzymes in the cocktail injected WT and CAR^-/-^ mice (left column) and the clearance of the associated probe drugs (right column) represented as area under the curve (AUC). Fed mice are represented in white and fasted mice in black bars. P-values represent the effect of fasting (*p*_fast_), genotype (*p*_gen_) or the interaction of both variables (*p*_int_) in the two-way ANOVA (n = 6 per group). Differences between groups as tested by posthoc Student’s t-test or nonparametric Mann Whitney U test are represented by * in case of the fed vs fasted groups (* = p≤0.05, ** = p≤0.01, *** = p≤0.001) and by ^b^ in case of the WT vs CAR^-/-^ groups (a = p≤0.05, *b =* p≤0.01)

Expression of nuclear receptors is presented in Fig 1. Cytochrome P450 mRNA expression was analysed as follows. To answer the first research question, mRNA expression of P450 enzymes in fed and fasted naïve (saline injected) WT and CAR^-/-^ mice was analysed with a two-way ANOVA with variables food and genotype. These data are presented in Fig 2. Since P450 expression is also known to be responsive to exposure of xenobiotics, such as the injection of the drug cocktail, relative data (to the respective saline injected groups) were analysed with a two-way ANOVA with the variables genotype and food. These data, calculated as fold increase to the matching saline control groups, are presented in Table 2. P450 mRNA expression in the fed and fasted WT and CAR^-/-^ mice that received the cocktail (not relative to saline groups to also show basal differences) was analysed with a two-way ANOVA with variables food and genotype. These data are presented in Fig 3, left panel.

**Table 2:** Substrate inducibility of P450 mRNA expression. Relative P450 mRNA expression in cocktail injected, fed and fasted WT and CAR-/- mice. Data are presented as the fold increase per group relative to the matching saline injected control group. Data were analysed by two-way ANOVA (n=6 per group).

	WT		CAR^-/-^		P-value ANOVA	
	Fed	Fasted	Fed	Fasted	genotype	interaction
PXR	0.09 ± 0.05	0.92 ± 0.11	1.21 ± 0.12	1.11 ± 0.11	NS	NS
Cyp1a2	0.83 ± 0.07	1.88 ± 0.73	0.87 ± 0.09	0.28 ± 0.06	**0.008**	**0.045**
Cyp2d22	0.83 ± 0.02	1.12 ± 0.07	0.92 ± 0.04	1.04 ± 0.09	NS	NS
Cyp3a11	0.75 ± 0.06	0.88 ± 0.18	1.22 ± 0.12	0.75 ± 0.13	NS	**0.029**
Cyp2c37	1.11 ± 0.23	1.36 ± 0.24	0.79 ± 0.13	0.45 ± 0.09	**0.013**	NS
Cyp2c38	0.84 ± 0.06	0.96 ± 0.08	0.80 ± 0.07	0.81 ± 0.16	NS	NS
Cyp2c65	1.02 ± 0.17	0.76 ± 0.13	0.77 ± 0.07	0.88 ± 0.19	**0.001**	NS

Plasma concentrations of each drug were measured in blood for pharmacokinetic analysis. Areas under the plasma concentration versus time curves (AUC) and clearance were determined by non-compartmental analysis using PKsolver [16]. AUC data were analysed by two-way ANOVA with genotype and food as the two variables. The AUC data are presented in Fig 3, right panel, next to the mRNA expression of the cytochrome p450 enzyme that is thought to mediate the metabolism of this specific drug.

For all data normal distribution was assessed by a Shapiro Wilk test on the residues of the ANOVA. In case of a skewed distribution, the data were ranked before performing the two-way ANOVA. Post hoc testing was performed by students t-test or Mann Whitney U nonparametric test.

## Results

### Effects of fasting on CAR, Cyp2b10 and PXR mRNA expression

CAR mRNA expression in CAR^-/-^ mice was on average 0.2% of that of WT controls (data not shown). Fasting increased CAR expression (*P*_ANOVA_≤0.001) in mice that were injected with the drug cocktail (Fig 1A). To determine CAR activation we measured the expression of the classic CAR target gene Cyp2b10. Fasting increased Cyp2b10 mRNA expression in saline treated WT, but not in CAR^-/-^ mice (Fig 1B).

PXR mRNA expression was not affected by either fasting or the absence of CAR in neither saline injected mice nor mice that received the drug cocktail (Fig 1C).

### Effects of fasting on the mRNA expression of P450 enzymes in WT and CAR^-/-^mice

Fasting decreased mRNA expression of Cyp1a2 and Cyp2d22 in saline injected WT and CAR^-/-^ mice. The decrease in Cyp1a2 was diminished, but not absent, in the CAR^-/-^ mice (Fig 2). In contrast, fasting increased the mRNA expression of Cyp3a11 and Cyp2c38 in saline injected WT and CAR^-/-^ mice. Fasting tended to decrease the mRNA expression of Cyp2c37 and Cyp2c65. Both basal and fasting-induced mRNA expression of Cyp2c37 were lower in the CAR^-/-^ mice compared to WT mice indicating a role for CAR in the basal expression of Cyp2c37. Cyp2c65 mRNA expression was lower in fasted CAR^-/-^ mice compared to fasted WT mice.

### Effects of substrate exposure on the mRNA expression of P450 enzymes in WT and CAR^-/-^mice

Injection of the cocktail did not affect Cyp1a2 mRNA expression in fed mice, whereas it increased Cyp1a2 expression in the fasted WT mice compared to saline controls (Table 2). In contrast, cocktail injection decreased Cyp1a2 mRNA expression in fasted CAR^-/-^ mice (*P*_genotype_ = 0.008, *P*_interaction_ = 0.045).

No effect on was seen on Cyp2d22 mRNA expression in fed and fasted WT and CAR^-/-^ mice.

Injection of the cocktail tended to decrease Cyp3a11 mRNA expression in the fed WT mice whereas it tended to increase the expression in the fed CAR^-/-^ mice (*P*_interaction_ = 0.029). No effect was observed in the fasted mice.

No effect on was seen on Cyp2c38 mRNA expression in fed and fasted WT and CAR^-/-^ mice.

Injection of the cocktail increased Cyp2c37 mRNA expression in fasted WT mice, whereas it decreased Cyp2c37 mRNA expression in fasted CAR^-/-^ mice (*P*_genotype_ = 0.013).

Injection of the cocktail had no effect on Cyp2c65 mRNA expression in fed WT mice, whereas it decreased Cyp2c65 mRNA expression in CAR^-/-^ mice (*P*_genotype_ = 0.001).

### Effects of fasting on the pharmacokinetics of the drug cocktail in WT and CAR^-/-^ mice

#### Cyp1a2-Caffeine

In the WT and CAR^-/-^ mice that received the cocktail, fasting decreased the mRNA expression of Cyp1a2. In agreement with this, fasting decreased caffeine clearance (as measured by an increase in exposure (area under the plasma concentration time curve (AUC)) (Fig 3). This effect was more pronounced in CAR^-/-^ mice.

#### Cyp2d22-Metoprolol

Fasting did not affect Cyp2d22 mRNA expression. Fasting, however, resulted in decreased metoprolol clearance in both WT and CAR^-/-^ mice. The absence of CAR aggravated the decrease in metoprolol clearance.

#### Cyp3a11-Midazolam

Fasting increased Cyp3a11 mRNA expression although this did not reach statistical significance in the CAR^-/-^ mice. Fasting had a small inhibiting effect on midazolam clearance in both WT and CAR^-/-^ mice. In addition, fed CAR^-/-^ mice had lower basal midazolam clearance.

#### Cyp2c38, Cyp2c37, Cyp2c65-S-Warfarin/Omeprazole

Fasting significantly increased Cyp2c38 mRNA expression in the WT but not in the CAR^-/-^ mice. Fasting did not affect Cyp2c37 mRNA expression in both WT and CAR^-/-^ mice while it decreased Cyp2c65 mRNA expression in both WT and CAR^-/-^ mice. The absence of CAR, however, resulted in markedly decreased expression of Cyp2c37 in the CAR^-/-^ mice.

Fasting decreased *s-*warfarin clearance in the WT but not in the CAR^-/-^ mice while fasting decreased the clearance of omeprazole in both the WT and the CAR^-/-^mice. Furthermore, both basal and fasting induced clearance of omeprazole were significantly lower in the CAR^-/-^ mice indicating that CAR is involved in the basal clearance of omeprazole.

### Summary of the results

1. Fasting had a clear differential effect on the expression of P450 enzymes. Fasting decreased the expression of Cyp1a2 and Cyp2d22, whereas it increased the expression of Cyp3a11 and Cyp2c38 mRNA (Fig 2). Basal expression of P450 enzymes did not differ between genotypes, except for Cyp2c37 which was significantly lower in CAR^-/-^ compared to WT mice.
2. Substrate exposure affects P450 mRNA expression. Injection of the cocktail tended to decrease mRNA expression of Cyp3a11 in fed WT mice whereas it tended to increase expression in CAR^-/-^ mice (Table 2). Differences in mRNA expression of Cyp1a2 and Cyp2c37 by substrate exposure were observed between fasted WT and CAR^-/-^ mice.
3. Fasting decreased the clearance of all drugs measured (Fig 3, right panel). The decreased clearance of caffeine and omeprazole were associated with the decreased expression of Cyp1a2 and Cyp2c37, respectively (Fig 3, left panel). The absence of CAR diminished the fasting-reduced clearance of *s*-warfarin and further aggravated the fasting-reduced clearance of omeprazole, metoprolol and midazolam.

## Discussion

In this study we show that the fasting-induced changes in the hepatic clearance of multiple widely prescribed drugs are largely independent of CAR, whereas the basal hepatic drug clearance of metoprolol, midazolam and omeprazole is CAR dependent.

Fasting induced the expression of CAR mRNA in cocktail injected mice, as seen before in rats [2] and mice [9]. CAR transcriptional activity can be regulated on both transcriptional and posttranscriptional levels. For example, phenobarbital activates CAR by stimulating translocation of CAR to the nucleus, thereby inducing its transcriptional activity [17]. Fasting induced activation of CAR was confirmed by measuring Cyp2b10, a prototypical CAR target gene, which was upregulated by fasting. The increase in CAR mRNA expression and activity is in agreement with earlier studies and involves the activation of nutrient sensing transcription factors HNF4 and PCG-1a [9]. In this study we did not observe an effect of fasting on PXR mRNA expression, as we have seen before in rats. It is likely that this finding represents a species difference.

The first aim or our study was to determine the effect of fasting on P450 mRNA expression in WT and CAR^-/-^ mice (displayed in Fig 2, left panel). Fasting decreased Cyp1a2 and Cyp2d22 mRNA expression, whereas it increased the expression of Cyp3a11 and Cyp2c38. Cyp2c37 and Cyp2c65 were not significantly affected by fasting but basal Cyp2c37 expression was lower in CAR^-/-^ mice indicating that CAR is important for the basal expression of this P450 isoform, or that this is due to a compensatory mechanism during development. The decrease in Cyp1a2 was attenuated in CAR^-/-^ mice, indicating that CAR is involved in the regulation of Cyp1a2, as was observed before [7]. The expression patterns we observed in the present study of Cyp1a2 (Cyp1a2 in rats), Cyp2d22 (Cyp2d2 in rats) and Cyp2c37 and Cyp2c38 (Cyp2c11 in rats) did not match our earlier observations in rats [2]. Cyp3a11 (Cyp3a2 in rats), however, showed comparable results; this P450 isoform is upregulated after fasting [2]. Since Cyp3a11 is a primary PXR target gene (7), it is likely that PXR instead of CAR is responsible for this response.

Since P450 enzymes are not only regulated by fasting, but also by exposure to their substrates, we determined the effect of the cocktail injection on P450 expression in WT and CAR^-/-^ mice (Table 2). Interestingly, none of the CYP isoforms was affected by the injection of the cocktail itself in WT fed mice, although this might be explained by the short time frame of 4 hours. However, in the absence of CAR, the cocktail induced-expression of some of the P450 isoforms (Cyp1a2 and Cyp2c37) in the fasted state and of Cyp3a11 in the fed state was attenuated, indicating that the substrate inducibility of these P450 enzymes is CAR-dependent. These results confirm that in addition to being a aryl hydrocarbon receptor (AhR) target gene [18], Cyp1a2 is also a primary CAR regulated gene as is also recently described by others [7].

The third and main aim of this study was to investigate whether the fasting-induced changes in hepatic drug clearance are mediated by CAR and to analyze mRNA expression of the P450 enzymes we hypothesize to be involved in the clearance of the drugs present in the cocktail.

CYP1A2 is responsible for caffeine clearance in humans. Caffeine clearance decreased upon fasting and this was not dependent on CAR. This decrease in clearance matched the decreased mRNA expression of Cyp1a2. In contrast, 36 hours of fasting increased oral caffeine clearance in healthy volunteers, in agreement with the increased Cyp1a2 expression in rats after fasting [2].

Metoprolol is predominantly metabolized by CYP2D6 in humans [10]. No effects of fasting on metoprolol clearance have been described [2]. Cyp2d22, an isoform that is abundantly expressed in mouse liver has been suggested to be the murine orthologue of human CYP2D6 [14]. Fasting decreased Metoprolol clearance, but this was not dependent on CAR. In addition, CAR^-/-^ mice showed decreased basal clearance of metoprolol. This was not correlated with Cyp2d22 mRNA expression.

Midazolam is predominantly metabolized by CYP3A in humans [10] and fasting does not affect the clearance of midazolam [2]. The murine ortholog is Cyp3a11 [19]. Fasting mildly increased midazolam clearance in contrast to the fasting-induced Cyp3a11 expression. In addition, the clearance of midazolam was further decreased in fed and fasted CAR^-/-^ mice. Midazolam can also be metabolized by Cyp2c in mice [20] which could explain the discrepancy between the expression of Cyp3a11 and midazolam clearance.

*S*-warfarin is predominantly metabolized by CYP2C9, whereas omeprazole is predominantly metabolized by CYP2C19 [10]. The CYP2C family is very large and complex, making it difficult to study the exact murine orthologs for these P450 isoforms. We chose Cyp2c37 and Cyp2c38, as these are the most abundant in mouse liver and Cyp2c65 [21]. Fasting decreased the clearance of *s*-warfarin in WT, but not in CAR^-/-^ mice (section *3*.*3*), indicating that this effect is CAR-dependent. Since Cyp2c38 mRNA expression increased upon fasting, it is likely that Cyp2c38 is not involved in *s*-warfarin clearance in mice. Fasting increased omeprazole clearance in both WT and CAR^-/-^ mice and basal clearance was decreased in CAR^-/-^ mice which is in accordance with the mRNA expression profile of Cyp2c37 and also partly with Cyp2c65. Since Cyp2c65 mRNA expression is very low compared to Cyp2c37 this suggests that Cyp2c37 is be the most important P450 isoform involved in xenobiotic metabolism in mouse liver and could be involved in *s*-warfarin and omeprazole clearance. In healthy volunteers, oral *s*-warfarin clearance was decreased after 36 hours of fasting [2], while no effect was observed for omeprazole.

It is clear from the above mentioned results, that the inter species differences between human, rat and mouse can make the translational interpretation of the results obtained from mice in this study to humans difficult for some P450 enzymes. The expression patterns of some P450 enzymes nicely correlate with the clearance curves of the drugs, in contrast to the patterns of Cyp1a2/caffeine and Cyp2c37/omeprazole. Whether these isoforms are indeed specific for these drugs in mice needs to be validated in *in vitro* models. In humans, no pharmacokinetic interactions between the probe drugs was observed, but this is not validated for mice.

In conclusion, we show that:

1. Fasting has differential effects on P450 mRNA expression.
2. The substrate-inducibility of Cyp1a2 and Cyp2c37 is CAR-dependent in the fasted state
3. CAR is important for the clearance of drugs by certain P450 enzymes but the fasting-induced changes in drug clearance are, except for *s*-warfarin, independent of CAR. The role of CAR in fasting induced changes in drug metabolism is limited.
4. To further investigate the involvement of other nuclear receptors in the regulation of P450 enzymes conditional knock out mice for these nuclear receptors (AhR, PXR, PPARα) could be used.

## Supporting Information

S1 File qPCR data file. (XLS)
